# Political alignment and project funding

**DOI:** 10.1007/s10797-022-09758-6

**Published:** 2022-08-23

**Authors:** Luisa Schneider, Daniela Wech, Matthias Wrede

**Affiliations:** Chair of Economics and Social Policy, Friedrich-Alexander Universität Erlangen-Nürnberg (FAU), Findelgasse 7, 90402 Nuremberg, Germany

**Keywords:** Project funding, Political alignment, Innovation policy, Regional policy, Intergovernmental relations, New public management, D72, R12, H77

## Abstract

We analyze the relationship between the party affiliation of politicians at different levels of government and the spatial distribution of funding for research, development and innovation projects. In particular, we are investigating whether more federal grants are being granted in Germany for projects in federal states whose government is led by the same political party as the responsible ministry at federal level. Our dataset contains detailed information on publicly funded projects in Germany in the period 2010–2019. Using a fixed-effects estimation approach, we find a link between grant allocation and party affiliation of funding for research, development and innovation projects, in particular smaller ones. For these projects, political alignment is associated with an average increase in public funding by almost 10,000 euro. Our results suggest that public funds for research, development and innovation projects could be used more efficiently than they are.

## Introduction

Economic growth and social returns are primary objectives of government investment. However, public investments and transfers serve not only the general interest but also the interests of specific groups. Driven by different motivations, politicians are likely to have an incentive to influence the allocation and spatial distribution of public investments and transfers. This also applies to the financing of research and development projects, provided that such projects bind highly qualified workers to the respective locations and have local multiplier effects. Questions frequently addressed in the literature are whether the location of core constituencies and swing voters or vote margins influence the distribution of transfers (see Dellmuth, Schraff, & Stoffel, 2017; Kauder, Potrafke, & Reischmann, 2016; Larcinese, Snyder, & Testa, 2013). In the same way, central governments might use transfers to support aligned subnational governments (see Brollo & Nannicini, 2012). Electoral concerns can be one reason for such *alignment biases*: Subnational governments who share the central government’s party are expected to offer support in upcoming elections. Moreover, central governments might especially support aligned subnational governments to reward loyalty and to push through the own political agenda on the local level. Firstly, this incentive is of relevance especially in Germany where many policy measures are implemented on the subnational level. The federal member states share state authority with the federation and are thus endowed with a variety of decision competencies. On the municipal level, the German constitution ensures a certain level of self-administration to facilitate independent decision making within local communities. Secondly, aligned governments might naturally have similar policy interests or tend to readily support central party decisions due to a given level of party discipline (Baskaran & Hessami, 2017; Curto-Grau & Zudenkova, 2018).

The international literature has addressed questions around potential alignment biases intensively, however mostly utilizes *intergovernmental transfers* in investigating the impact of such political compounds. While recent studies suggest that higher-tier governments tend to distribute intergovernmental transfers in favor of lower-tier governments with the same political affiliation (see Baskaran & Hessami, 2017; Bracco, Lockwood, Porcelli, & Redoano, 2015; Brollo & Nannicini, 2012), only little is known on the impact of political alignment on *project funding* in the highly relevant field of research and innovation.

We extend previous studies by examining the effect of political affiliations between federal and subnational governments on research and development funding, which has, to our knowledge, not yet been considered in this regard. Our dataset contains detailed information on publicly funded research and development projects in Germany over the period 2010–2019. Since a large number of recipients receive funding several times, the identifying variation occurs between projects from the same grant recipient. We thus analyze whether recipients’ projects are granted higher amounts in periods where the state they are located in is politically aligned with the federal government. This allows us to analyze whether federal states which are ruled by the same political party as the federal ministry which distributes the grant, benefit from this political connection. Since state governments only benefit indirectly from project grants, finding a significant impact could be of particular interest and would suggest that distortions might even exist in areas where one would not expect them in the first place. It is particularly important to analyze and optimize the allocation of funds, since in Germany high and increasing public expenditures are made for the support of research and innovation. Having spent 3% of GDP on research and innovation in 2018, Germany has already reached one goal of the Europe 2020 strategy with a new target set to 3.5% until 2025. This corresponds to an increase of government investments in this field by around 70% between 2005 and 2017 (Bundesministerium für Bildung und Forschung, 2019, 2020). The importance of research and innovation in previous years’ budgets becomes even clearer when compared to infrastructure-related investments, which amounted to just 1.7% of GDP in 2018 (Bundesministerium für Wirtschaft und Energie, 2020). Governmental research and innovation investments are one key element in fostering regions’ competitiveness and achieving equal living conditions (Bundesministerium für Bildung und Forschung, 2020). Moreover, research has shown that such investments yield positive externalities for recipient regions (see Kang et al. (2019) and Tingvall and Videnord (2020), for instance). Distributing funds based on political rather than normative criteria is likely to result in a misallocation of resources which questions the efficiency of concerned investment programs. In the long run, misallocations might affect economic growth and cultivate disparities between regions (Kitsos & Proestakis, 2021; Asher & Novosad, 2017). Moreover, distortions due to political alignment are likely to intensify perceived corruption which in turn can lower voter turnout and overall trust in democratic institutions.

Following the existing literature on intergovernmental transfers, we evaluate whether political alignment impacts the geographical allocation of governmental funding for research, development and innovation projects across German states. In contrast to the majority of studies, we use data on direct project funding instead of intergovernmental transfers in the specific field of funding for research, development and innovation. Novel insights can be expected in this regard for several reasons. (1) We transfer the international evidence on alignment effects to funding for research, development and innovation projects. Ensuring effectiveness and equal opportunities regarding project funding is especially necessary with regards to this field’s relevance and competitiveness. (2) Direct project funding differs from intergovernmental transfers in that the recipients are not directly linked to subnational governments and political parties. The fact that lower-tier governments are not supported directly and that the additional funds are only indirectly credited to the lower-tier government by the voters can have a positive or negative effect on the political distortion of the allocation of funds. On the one hand, the incentive for the political leadership of the Federal Ministry to support regions that are led by governments with the same political orientation could be weaker, since the governments in the recipient regions benefit less at the ballot box. On the other hand, the project funding makes it possible to disguise the party-political context, which could have an incentive-enhancing effect. Our hypothesis is that political alignment ultimately has a non-negligible effect on project funding.

In line with this hypothesis, we find that political affiliation between receiving state and giving federal government (ministry) is associated with a substantial increase of the funding amount of projects in the respective states, in particular for smaller projects. On average, it takes three months for the political alignment to have a significant influence on the level of project financing. We find that the effects do not occur for state governments affiliated with other parties in the governing coalition at the federal level or with other parties on the same political spectrum. At the aggregate level, we find that political affiliation only has significant effects on the amount of funding per project, but neither on the likelihood of any funding nor the number of projects, probably reflecting that the political influence is primarily in the setting of the project budget.

The remainder of the paper is structured as follows. Section 2 gives an overview on the existing international empirical literature we contribute to. In Sect. 3, we explain the research and development grant system as well as parts of the political landscape in Germany that are relevant for our study. In Section 4, we describe our dataset and show some descriptives. In Sect. 5, we describe our identification strategy and the results including robustness checks. Section 6 concludes.

## Literature

Our paper contributes to three broader strands of literature. The first strand is the literature on the political economy of (inter)governmental transfers. The role political motives play in the distribution of governmental transfers has been addressed in several ways. Besides effects of political alignment, a large body of empirical literature investigates pork barrel spending intended to benefit the home districts of Congress members.^1^ Additionally, several recent studies addressed the relationship between public investment and the location of core supporters.^2^ Dixit and Londregan (1995) provide theoretical frameworks which show that redistribution is impinged by political determinants, Brollo and Nannicini (2012) extent theory by specifically addressing political alignment. A large body of empirical literature exists on the USA. While Levitt and Snyder (1995) do not find significant political alignment effects on federal grants flowing to districts in general, other studies’ results suggest that political alignment does matter for the distribution of US government grants (see Grossman (1994), Larcinese et al. (2006) and Berry et al. (2010), for instance). Furthermore, many studies on the USA show variations in political alignment effects for different types of grants and political parties (Albouy, 2013; Young & Sobel, 2013; Reingewertz & Baskaran, 2020). For Brazil, Brollo and Nannicini (2012) compare municipalities in which the candidate affiliated to the presidential coalition at federal level narrowly won the local elections with municipalities in which the affiliated candidate narrowly lost. They show that mayors aligned with the president receive major infrastructure transfers after being elected in the two years leading up to the next local elections. Their results suggest that there is no alignment in the first two years of the mayor’s tenure. Sakurai and Theodoro (2020) show that the effect of political alignment between mayors and the federal government is substantial for capital transfers, but negligible for other current transfers. Moving to Europe, Solé-Ollé and Sorribas-Navarro (2008) show that alignment with upper-tier governments leads to around 40% more capital grants for Spanish municipalities. Bracco et al. (2015) find similar results for Italy. Moreover, alignment affects re-election probabilities and effects are more pronounced in the run-up to municipal elections.^3^ Baskaran and Hessami (2017) investigate the impact of political alignment on budget support transfers from the German federal state of Hesse. Results show that local municipalities who share Hesse’s political party only receive more grants if the local support for the state government is strong. Kemmerling and Stephan (2002) evaluate infrastructure investment grants directed from federal states toward German cities. They find that the political affiliation of the local city government and the state government is an important factor in explaining the allocation of grants to 87 studied cities. Finally, Bury et al. (2020) extend studies looking at direct granting relations by investigating budget support transfers to municipalities for which expense federal and state governments are involved. They demonstrate that directly elected members of the federal parliament channel more grants to their constituencies if their party governs the federal and state level governments.

Furthermore, we contribute to the broader literature on the effects of political alignment. For instance, Asher and Novosad (2017) find that local regions in India which are governed by the ruling party experience an increase in local economic growth compared to opposition-governed regions. Regarding the politicization of the bureaucracy, various authors show that good political connections increase the likelihood of employment, promotion and higher wages in the public sector.^4^ Palus and Yackee (2016) show that US state agency heads receive less freedom in major policy, administrative or budget decisions when their partisanship is aligned with the governor or state legislature. Concerning the effects of political alignment on voting behavior, Solé-Ollé and Sorribas-Navarro (2008) indicate that allocating grants to aligned governments does buy local support in the case of Spanish municipalities. Garofalo et al. (2020) suggest that Argentinian voters also tend to support candidates aligned with the president’s party in expectation of benefiting in future monetary transfers.

Finally, we contribute to the literature exploring (the allocation of) grants in the field of research and development. While a large body of research evaluates the effectiveness of grants in this field,^5^ our focus is the allocation and potential politico-economic determinants therein. For Germany, Aschhoff (2010) shows that the distribution of subsidies is determined by prior grant receipt and Cantner and Kösters (2012) find that, for start-ups, subsidies are given out based on high quality inventions and innovative business ideas. The literature investigating political motives in the distribution of funding for research, development and innovation projects is scarce. Payne (2003) utilizes federal research funding to US universities to demonstrate that appropriations committee members distribute grants based on constituents and personal interests. Being affiliated with the party which is in power in the congressional chamber matters, too. Hegde and Mowery (2008) support the findings regarding congressional appropriations committee members for federal funding for biomedical research. Exploring scientific research grants in Germany, Grimpe (2012) finds that normative criteria determine the allocation of part of the studied grants. However, for government grants, this relationship could not be established, which suggests that political factors might play a role in this context.

## Institutional background

### The German federal system

Within Germany’s federal system, the federation and the 16 federal states share state authority. Each federal state is governed by a state prime minister (“Ministerpräsident/in”) who is elected by the state parliament. The states are endowed with own competencies regarding legislation, administration and jurisdiction. State competencies lie in many different policy fields, such as cultural issues, school and education policy, municipal affairs (organization rights for cities, municipalities and counties), police law and public health infrastructure. Moreover, state prime ministers can contribute to political decisions on the federal level through their participation in the federal council (“Bundesrat”). Federal and state competencies are defined in the German basic law (particularly articles 71 to 74). For an overview on administrative federalism in Germany, see Behnke and Kropp (2021). In general and in terms of decisions within the federal council, it is likely that prime ministers often face a tradeoff between representing their states’ interests and giving into party discipline (Plöhn & Steffani, 1994).

### Federal research and innovation funding

The German federal government offers direct project funding in the form of (see Bundesministerium für Bildung und Forschung - Referat Informationstechnik, 2020). The provision of these grants is administered and financed by several federal ministries, namely the Ministry of Economic Affairs, the Ministry of Transport, the Ministry of Environment, Nature Conservation and Nuclear Safety, the Ministry of Food and Agriculture and the Ministry of Education and Research.^6^ Potential recipients include German industrial companies, universities and institutions of higher education or other (federal) institutions engaged in research and innovation activities.^7^ The responsible ministries make their program proposals publicly available via the federal gazette or their websites. Program proposals mainly encompass information on funding purpose, target group, prerequisites, amount of assistance, time frame and application and admission process.^8^ While project management organizations are in charge of the programs’ technical and administrative coordination, the final admission decision is made by the executing ministry (Bundesministerium für Bildung und Forschung - Referat Grundsatzfragen von Innovation und Transfer, 2021). Since programs are not completely identical in their application and approval processes, the exact time structure from project application through admission to project start is not directly traceable, which leads us to also looking at lags.

## Data and descriptives

In this section, we describe the dataset, our treatment definition, and provide some descriptive statistics.

### Data

To answer our research question, we make use of a publicly accessible database provided by the federal government. The so-called *funding catalogue* documents projects in the fields of research and innovation that were accepted for government funding within the offered programs (Bundesministerium für Bildung und Forschung - Referat Informationstechnik, 2020). The dataset contains information on grant recipient, location of the recipient (municipality level), project duration and the amount of assistance granted for the full project length. We observe around 107,000 funded projects between 2010 and 2019. Grant recipients are primarily universities, research institutes and private companies and many recipients obtain funding several times. We leave out the most recent projects starting in 2020 to avoid measuring any special effects due to the Covid-19 pandemic. Projects are incorporated into the database 60 days after approval. The database provides rather detailed information on granted projects, Table 10 in the Appendix gives an overview on the database’s structure.

**Table 1:** Funding 2010-2019 (million euro): Funding Catalogue vs. Federal Report on Research and Innovation

	Funding Catalogue	Federal Report on Research and Innovation
BMWi	8772.05	9805.50
BMBF	34,737.71	32,820.70

Notes: Funding by the Ministry of Economic Affairs (BMWi) and the Federal Ministry of Research and Education (BMBF). *Data Sources:* Funding Catalogue (Bundesministerium für Bildung und Forschung - Referat Informationstechnik, 2020) and Federal Report on Research and Innovation (Bundesministerium für Bildung und Forschung, 2020)

One drawback of our dataset is that it does not constitute a complete coverage of all accepted projects. Each ministry decides which projects enter into the database which might raise concerns whether certain projects are systematically excluded from our database. To cross-check our numbers and get a tendency on how many projects might be missing in our dataset, we looked at the actual amount expended by the government on funding for research, development and innovation projects, which numbers are readily available from the federal report on research and innovation and its accompanying database (Bundesministerium für Bildung und Forschung, 2020). While the federal report documents actual expenditures during any given year, the funding catalogue includes expenditures for the whole lifetime of a project (documented in the first year of a project). For instance, if a project that starts in 2019 then the whole amount of assistance for the lifetime of this project will be documented in the funding catalogue, whereas in the federal report only the fraction that was actually given to the project in 2019 will be shared. Numbers are thus not comparable on a yearly basis, so we compare the sum of the amounts of assistance from 2010 to 2019. See Table 1 for the aggregated comparison, where we show numbers (in million euro) for the federal ministry of economic affairs (BMWi) in the top row and the federal ministry of research and education (BMBF) in the bottom row. For the BMWi, the numbers indicate that the funding catalogue actually covers around 90% of actual expenditures, so the number of projects not published in the funding catalogue does not seem to be remarkably high. Comparing the numbers for the BMBF yields a similar picture, where the difference between funding catalogue and actual expenditures lies at around 6%. The lower numbers for the federal report can be explained by the differences in documenting the amounts of assistance between the two databases, meaning the timing when project expenditures are documented. Unfortunately, the federal report only reports numbers on an aggregated level (per ministry or state), so we cannot deduce any information on the type of projects that are excluded from our dataset. However, from this first comparison, we can conclude that there is no massive exclusion of projects. If ministries did not publish certain projects for tactical reasons, for example, because political incentives play a role, and we still estimate an effect, this effect would be underestimated. It would also be unproblematic if certain projects were omitted only by chance, since in this case the estimation results would still be unbiased.

A big advantage of the database we use is the very large number of observations—more than 100,000. A closer look at the database reveals that there are very few projects with extremely large amounts of assistance. The top five largest values are around 500 to 4000 times as large as the average amount of assistance grant recipients get with the maximum value reaching over 2 billion euro. Table 11 in the Appendix shows the top ten largest projects. It is very plausible that the possibilities and incentives for politically motivated interventions, but also compliance rules, depend on the size of the project. The Federal Office of Administration − Competence Centre (Major) Project Management provides a suitable way of addressing this question. Projects are classified according to project size − small projects are defined by a maximum volume of 2 million euro, medium-sized projects are larger than 2 million euro and have a maximum volume of 10 million euro, major projects are larger than 10 million euro, and mega projects are larger than 100 million euro (Kompetenzzentrum (Groß-)Projektmanagement, 2020). Table 12 in the Appendix provides a more detailed overview of the project classification according to project size. In our regressions, we account for project size accordingly. Table 2 shows the number of projects we include in our analyses, as well as shares and expenses by category. In total, there are around 107,000 projects in the funding catalogue for the ten-year-period from 2010 to 2019. Excluding observations with amounts of assistance below 200 euro reduces the sample by around 200 projects. The exclusion of projects where grant recipients and executing entities are not in the same federal state, which we explain in the next subsection, leads to a reduction of the sample size by around 8000 observations. The huge majority of the close to 99,000 projects in our sample are small projects (almost 96,000). There are around 2600 medium-sized projects and nearly 500 large projects. Due to the large number of small projects, they make up about 50% of the total expenditure, and medium-sized projects another 21%.

**Table 2:** Sample

	# Projects	In %	% of total expenditure
Projects in database 2010–2019	107,019		
Projects funding amount $\ge$ 200 euro	106,803		
Our sample	98,870		
Small projects	95,782	96.88%	50.51%
Medium-sized projects	2614	2.64%	20.90%
Large projects	474	0.48%	28.59%

Notes: our sample: projects with funding amounts $\ge$ 200 euro + executing entity and grant recipient are located in the same state. Classification of projects: small projects $\le$ 2 million euro, medium-sized projects $\le$ 10 million euro, large projects > 10 million euro (which include mega projects, > 100 million euro, representing less than 0.02% of all projects)

An important feature of the funding is the unequal distribution among the recipients. A total of 23,877 recipients benefit from the grant, but 13,522 recipients receive only one grant. The mean value of the number of funded projects per recipient is 4.14. Six recipients even receive funding for 700 or more projects. In terms of the number of projects, RWTH Aachen University is at the top with 1264 projects, followed by other technically oriented universities, spread across different federal states with a focus on the federal states of Baden-Württemberg and Bavaria. Project funding captured in our dataset varies significantly over time and space. The number of funded projects per month varied over our observation period of 120 months between at least 191 and at most 2098 with an average of 823.92 projects and a clear upward trend. The project funding is widely spread across the federal states and as a whole roughly follows the distribution of the population (see Table 13 and Figure 2 in the appendix). Nevertheless, there are substantial differences in the number of funded projects per inhabitant, which is not surprising given the differences in sectoral structures and productivity.

### Treatment

Our treatment indicator is equal to 1 if the responsible federal minister and the state prime minister have the same party affiliation at the time of the start of the project. In our sample, this is the case if the federal minister and the state prime minister are both either from the Christian democratic union (CDU)/Christian social union (CSU) or from the social democratic party (SPD). In our benchmark analysis, we do not differentiate between the CDU and the CSU, because the CSU is only active in Bavaria (“sister party” of the CDU), the CDU, however, in all other federal states and both parties form a parliamentary group in the German federal parliament (“Bundestag”). Later we will also consider the two parties separately. The treatment indicator is equal to 0 for all other combinations of party affiliations between federal ministers and state prime ministers. During the period under review, federal ministers and prime ministers of other political parties were also in office (the free democratic party (FDP), the greens, and the left party); between 2010 and 2019, however, there was never a period in which a federal minister and a state premier belonged to the same party, with the exception of the CDU / CSU and the SPD.

The exact coding of the treatment indicator is as follows: If there is a change in the party affiliation of the federal minister or the state prime minister in the course of a month, the indicator changes its value on the 1st of the following month; a project usually starts at the beginning of a month. We have information on the beginning (and the end) of the project duration; data on the time of approval is unfortunately not available. However, the following two facts allow us to set up our timing structure. It is indicated in the funding catalogue that projects are included in the database 60 days after approval. On top of that, we have the following information on the timing structure: The beginning of the project duration of the most recent projects included in the database ranges from around several months to half a year in the future, and in very few cases, up to one year. From this time frame, one can infer that it is reasonable to assume that projects usually start rather quickly after they were approved. To account for potential delays between the approval of a project and the beginning of the project duration, in one specification, we also analyze lags of the treatment indicator.

Our dataset makes it possible to distinguish between the grant recipient and the executing entity and their respective location. In order to answer our research question as to whether the prime ministers benefit from the same party membership of the federal minister, the respective federal state must actually benefit from the funding by the federal ministry. To make sure that this is the case in our analysis, we only include funded projects in our sample where the grant recipient and the executing entity are located in the same federal state. If the executing agency is located in a different federal state than the grant recipient, which may be the headquarters of a company, for example, it is difficult to determine whether the federal state in which the executing agency is located, or rather the federal state in which the grant recipient is located, benefits from the support. One could argue generally in both directions—the region of the executing entity could benefit from increasing investment and also employment, but it might also be beneficial to the grant recipient, especially in politico-economic terms. Greater support for the region can be seen as a sign of particular political commitment and success on the part of state politicians and increases their chances of re-election. For these reasons, we exclude all projects where the grant recipients and the executing entities are not located in the same federal state. Since, in most cases, grant recipients and executing entities are actually located in the same federal state, we exclude only around seven percent of observations. Lastly, our focus on the political ties between the federal and state governments is advantageous: Since the supported regions that we are looking at, the federal states, are rather large, the positive economic effects will most likely be limited to the region in question, so that regional spillovers are of relatively little importance, which would be different if we examined smaller regions. In addition to spatial spillovers, the characteristics of the grant recipients and the German electoral system as well as the limitations of our dataset make it difficult to analyze relationships at the community or constituency level. Not only the members of the Bundestag elected directly in the constituencies—via the so-called first vote—but also most of the members elected via the lists of the parties—via the so-called second vote—also ran in constituencies and therefore have a close connection to the constituencies. Most constituencies have members of various parties in the Bundestag. In addition, large cities, in which many projects are typically located, are divided into several constituencies and large beneficiaries often have several locations in one city—possibly distributed across the constituencies. In our sample, there are only 14,415 projects in municipalities that are only represented by one or more directly elected members of the same party and not by other directly or indirectly elected members of other parties in the Bundestag. In addition, for 5811 projects, the beneficiaries and sponsors are in the same federal state, but not in the same municipality. Furthermore, our dataset only contains information about the municipality, but not the exact address, so that we cannot assign the projects in large cities to a constituency. Finally, we cannot allocate 4045 projects to a constituency because the allocation from municipality to constituency is based on the municipality structure at the time of the federal election, while the information on the projects is based on the current municipality structure. Any assignment of constituencies to projects based on area, population or employment would lead to measurement errors. Due to these limitations, we have refrained from a direct analysis of the members of parliament. In a robustness analysis, however, we include state-election-periods fixed effects and, alternatively, state-ministry-election-periods fixed effects, which should at least partially capture the influence of the members of parliament.

### Descriptive statistics

Table 3 provides descriptive evidence on the magnitude of the assistance. The average amount of assistance of all projects in our sample lies at around 500,000 euro, but the median value is only below 200,000 euro. A large majority of projects have a volume below 500,000 euro and only few projects have a volume above 1 million euro. Medium-sized projects have an average volume of almost 4 million euro, the median value lies at around 3.3 million euro. For large projects, the mean value is around twice as high as the median value − approximately 30 million euro in comparison to 15 million euro (Table 3). The extreme differences in the size of the projects seem to make a separate analysis according to project size reasonable as already explained in the previous section. Even within the category of small projects, there is considerable variation − a huge majority of projects below 1 million euro and only few projects above 1 million euro; however, in order to avoid an arbitrary classification, we stick to the official categorization of projects according to project size given by Kompetenzzentrum (Groß-)Projektmanagement (2020). Finally, we only include projects with an amount of 200 euro or more in our regressions.^9^

**Table 3:** Summary statistics: amount of assistance

	Minimum	1st quantile	Median	Mean	3rd quantile	Maximum
All projects	200	50,008	173,418	504,794	370,492	2,049,099,650
Small	200	50,000	163,439	263,172	342,226	2,000,000
Medium	2,000,311	2,498,698	3,300,318	3,990,514	4,992,032	10,000,000
Large	10,004,584	11,838,652	15,000,000	30,106,885	23,171,498	2,049,099,650

Notes: small projects $\le$ 2 million euro, medium-sized projects $\le$ 10 million euro, large projects > 10 million euro

**Fig. 1:**
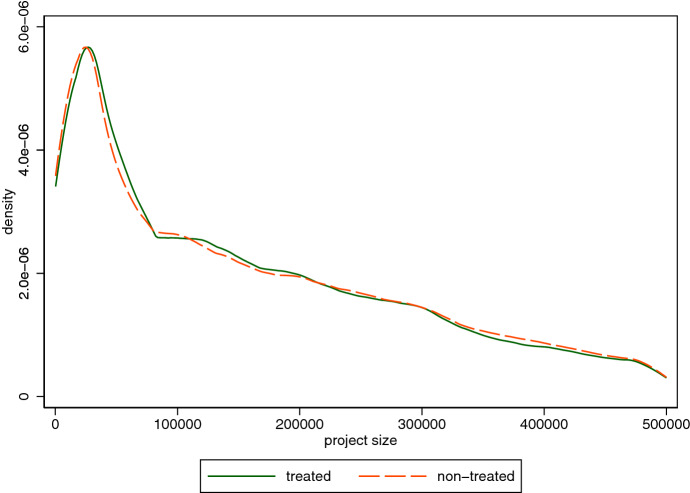
Distribution of project sizes—treated vs. non-treated projects ($\le$ 500,000 euro). Notes: illustration of the (smoothed) distributions of “treated” (solid green line) and “non-treated” (dashed red line) projects up to a volume of 500,000 euro. Treated projects refer to projects that were granted in a federal state during a time when the respective state prime minister and the respective federal minister had the same party affiliation. Otherwise, projects are classified as non-treated projects (Color figure online)

**Fig. 2:**
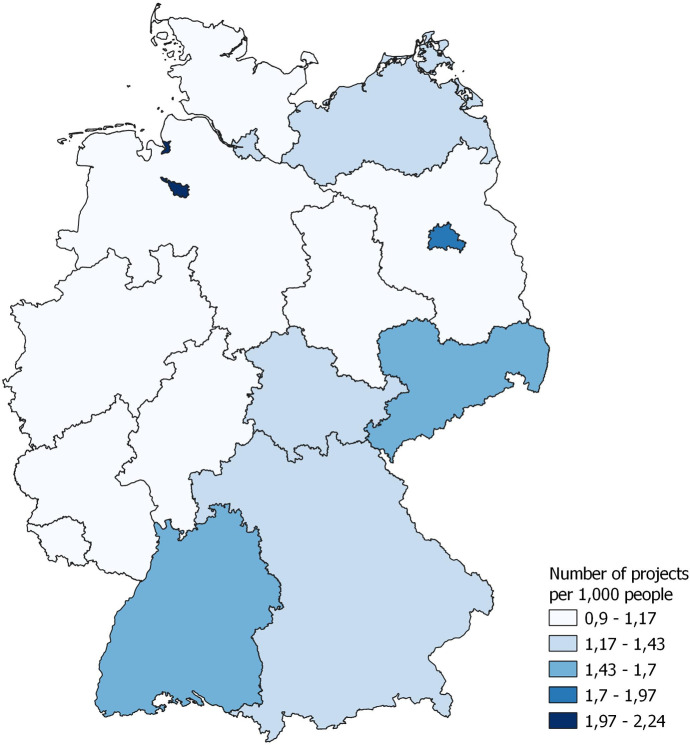
Number of projects (per 1000 people) across states. Notes: illustration of number of projects per 1000 people granted in each federal state in the observation period 2010 to 2019. We use the number of inhabitants from December 2018. *Data Sources:* The number of projects is taken from the Funding Catalogue (Bundesministerium für Bildung und Forschung - Referat Informationstechnik, 2020); the number of inhabitants per state is collected from the federal statistical office

Figure 1 illustrates the (smoothed) distributions of “treated” and “non-treated” projects up to a volume of 500,000 euro. Treated projects refer to projects that were granted in a federal state during a time when the respective state prime minister and the respective federal minister had the same party affiliation; otherwise, projects are classified as non-treated projects. Firstly, the figure demonstrates the rather asymmetric distribution of project size below 500,000 euro. Secondly, the distributions of treated and non-treated projects are rather similar; but for very small projects, the share of non-treated projects is higher than that of treated projects, whereas for slightly larger projects it is the other way around.^10^ Table 4 shows the number of treatment changes federal states experienced in our observation period. New federal governments came into office after federal elections in 2013 and 2018.^11^ Apparently, the majority of treatment changes occurred in connection with federal elections, as shown in the last row. However, we also observe a significant number of treatment changes in years without federal elections. Hence, variation in the treatment indicator is not limited to only a few years, but generally occurs in many of the years of our observation period. Moreover, the last column of Table 4 demonstrates that we observe treatment changes for every state as well. For 10 federal states, changes of the treatment status occurred twice or three times. Only for two federal states, the treatment status changed 12 times (including simultaneous changes for different ministries).

**Table 4:** Number of treatment changes

State	2010	2011	2012	2013	2014	2017	2018	Total
Baden-Württemberg	0	4	0	0	0	0	0	4
Bavaria	0	0	0	1	0	0	1	2
Berlin	0	0	0	2	0	0	1	3
Brandenburg	0	0	0	2	0	0	1	3
Bremen	0	0	0	2	0	0	1	3
Hamburg	0	4	0	2	0	0	1	7
Hesse	0	0	0	1	0	0	1	2
Lower Saxony	0	0	0	6	0	0	1	7
Mecklenburg-Vorpommern	0	0	0	2	0	0	1	3
North Rhine-Westphalia	4	0	0	2	0	5	1	12
Rhineland-Palatinate	0	0	0	2	0	0	1	3
Saarland	0	0	0	1	0	0	1	2
Saxony	0	0	0	1	0	0	1	2
Saxony-Anhalt	0	0	0	1	0	0	1	2
Schleswig-Holstein	0	0	4	2	0	5	1	12
Thuringia	0	0	0	1	3	0	0	4
Total	4	8	4	28	3	10	14	71

Notes: number of treatment changes across states and years. There are no treatment changes in 2015, 2016 and 2019

## Empirical analysis

To address our research question, we apply a fixed-effects approach.

### Identification strategy

First, we estimate the following four-way-fixed-effects regression:

1 $$\begin{aligned} Y_\mathrm{pkijt} = \beta {X_\mathrm{ijt}} + \eta _k + \gamma _i + \delta _j + \lambda _t + \epsilon _\mathrm{pkijt}\,, \end{aligned}$$

where *X* is a binary treatment indicator that takes on the value 1 if the federal minister and the state prime minister have the same party affiliation, and $\beta$ is our main coefficient of interest. *Y* denotes the outcome variable, amount of assistance, which we consider in logarithms to better account for its distribution. Our analysis is at the project level, denoted by the index *p*. Grant recipient-fixed effects are indicated by $\eta _k$, state-fixed effects by $\gamma _i$, ministry-fixed effects by $\delta _j$ and month-times-year-fixed effects by $\lambda _t$. The error term is denoted by $\epsilon _\mathrm{pkijt}$. In our analysis, we have 23,877 grant recipients, 16 states, 5 ministries and 120 months. Including these fixed effects allows us to control for general variations in the amount of assistance over time, across grant recipients and federal states and between ministries.

The identifying variation occurs between projects from the same grant recipient. We thus analyze whether a recipient’s projects are granted higher amounts if they are granted in a federal state during a period where the respective state prime minister and the respective federal minister are affiliated with the same party. Due to the large number of grant recipients with several funded projects, we can use recipient-fixed effects and thus control for the specific eligibility of recipients and to a certain extent also for properties of the projects—such as technology orientation. Due to the month-times-year-fixed effects, we take into account, inter alia, any trends in the allocation of funds and the effects of special events such as the elections as well as seasonal patterns. Stable differences in the budgets are absorbed with the ministry-fixed effects. The state-fixed effects make it possible, among other things, to take time-invariant differences in the sectoral structure and productivity between the states into account.

Secondly, our baseline estimation examines only the impact of belonging to the same party on project funding in the same month. However, it is quite plausible that, especially after the change of minister at federal level, it will take some time before new programs and guidelines are developed and people are exchanged. We therefore expect the effects of the change to be greatest after a few months. To test this hypothesis, we examine the effect of the timing of the change into treatment. The respective estimation equation is:^12^

2 $$\begin{aligned} \begin{aligned} Y_\mathrm{pkijt} =~&\beta _{-H-1} {{\tilde{\Delta }}_{i,j,t-H-1}} + \sum _{h=1}^{H} \beta _{-h} {\Delta _{i,j,t-h}} + \beta _0 {\Delta _{ijt}}\\&+ \sum _{h=1}^{B} \beta _{h} {\Delta _{i,j,t+h}} + \eta _k + \gamma _i + \delta _j + \lambda _t + \nu _\mathrm{pkijt}\,. \end{aligned} \end{aligned}$$

The binary right-hand-side variable, $\Delta _{i,j,t}$, indicates a change from different parties to same party for state *i*, ministry *j*, and period *t*. ${\tilde{\Delta }}_{i,j,t-H-1}$ summarizes changes into treatment in period $t-H-1$ and earlier. The number of lags, *H*, and leads, *B*, may differ. The omitted category captures all changes from different parties to same party that occur later than $t+B$ or never. Since we choose *H* and *B* so small that in our dataset there is no state-ministry pair with more than one treatment change in a period of length $B+H+2$, at maximum only one of the change variables $\Delta$ and ${\tilde{\Delta }}$ is different from zero. Month-, grant-recipient-, state- and ministry-fixed effects are still included, and $\nu _{pkijt}$ denotes the error term.

Thirdly, since we can neither observe potential nor submitted but rejected applications, our analysis at the project level suffers from a selection of particularly promising projects in the sample. If our hypothesis that party-political closeness between the ministry and the state government increases the chances of success is correct, then this closeness stimulates the submission of more projects and reduces the likelihood of rejection. We therefore presumably underestimate the real impact of political proximity. In order to determine the overall influence of political proximity, we also aggregate projects monthly for all federal state-ministry combinations leading to the three-way-fixed-effects estimation equation

3 $$\begin{aligned} Y_\mathrm{ijt} = \beta {X_\mathrm{ijt}} + \gamma _i + \delta _j + \lambda _t + \mu _\mathrm{ijt}\,, \end{aligned}$$

where $\mu _\mathrm{ijt}$ indicates the error term. $Y_\mathrm{ijt}$ is the total amount of assistance for state *i* from ministry *j* in month *t*. Month-times-year, state- and ministry-fixed effects are still included. The aggregated funding amounts summarize the impact on submissions, rejections and funding amounts. On the other hand, due to the reduction in the number of cases associated with aggregation, the effects are less precisely estimated, and there are also many state-ministry-month combinations without a single project.

### Estimation results at the project level

Table 5 shows the results of our main specification based on Eq. (1). At first we only look at small projects; later we check to what extent the results change when larger projects are included. The dependent variable is the log of the funding amount and the treatment is defined as same party, where we consider the two “sister parties” CDU and CSU as one party. We include month-times-year-, grant recipient-, state- and ministry-fixed effects as explained before; standard errors are clustered at state level, which includes the combination of federal states and ministries, i.e., the level on which the variation in our treatment indicator occurs. To account for the small number of clusters, using boottest developed by Roodman et al. (2019) we apply wild bootstrapping to calculate standard errors and *p* values.^13^

The treatment coefficient of Model (1), which does not include further covariates, is positive and statistically significant on the 5% level. This implies that the amount of assistance for projects in treated states is higher than of those located in untreated states. A link between political alignment and grant allocation exists for small research and innovation projects: federal ministers tend to favor state prime ministers belonging to the same political party. Our estimate indicates that treatment increases financial support by 4.11%. From the corresponding linear regression, we obtain that the average absolute treatment effect equals 9534.52 euro. These numbers are clearly not negligible.

Models (2–4) extend our baseline specification by including alternately, but also jointly the dummies home constituency and joint project. The treatment coefficient is marginally insignificant in Model (2), but significant at the 5% and the 10% level in Models (3) and (4), respectively. The binary variable home constituency controls in Models (2) and (4) for federal ministers having their home constituency in the respective federal state, the variable joint project in Models (3) and (4) for projects being part of so-called joint projects that consist of several projects. We control for the constituency because, as mentioned in the literature review, many studies found that politicians favor their home constituencies.^14^ However, we specifically address projects in which several project partners apply for a project together (joint projects). Although each project is still allocated an individual amount of assistance, the decision on whether a project is approved or not affects all project partners. While the coefficients for the home constituency dummies are positive, but comparably small and completely insignificant, those for joint projects are highly significant, comparably large and also positive. The treatment coefficient remains consistent. This indicates that the link between party affiliation and grant allocation is still prevalent when controlling for home constituencies and joint projects. Our results do not provide evidence on federal ministers favoring the federal states where their home constituencies are. For joint projects, the amount of assistance each project gets is considerably larger, which might be caused by higher quality or more intense lobbying.

We also check whether our main result is robust to alternative sets of fixed effects. Table 14 in the appendix shows that neither dropping recipient-fixed effects nor substituting state- and ministry-fixed effects with state-ministry-fixed effects changes the coefficient of the treatment variable substantially, which, however, is weakly insignificant in the absence of recipient fixed effects. To capture the influence of members of parliament at the state level, we add a model that includes fixed effects for the three election periods at the federal level (2009–2013, 2013–2017, 2017–2021) combined with state fixed effects and a model with state-ministry-election-periods fixed effects. A significant treatment effect is retained in both models; the inclusion of state-ministry-election-periods fixed effects even increases the measured treatment effect.

**Table 5:** Estimation results for small projects

	(1)	(2)	(3)	(4)
Treatment	0.0403	0.0393	0.0404	0.0392
	(0.0459)	(0.1071)	(0.0290)	(0.0824)
Home constituency		0.0057		0.0072
		(0.8306)		(0.7529)
Joint project			0.5536	0.5536
			(0.0000)	(0.0000)
*N*	82,249	82,249	82,249	82,249
$\hbox { R}^2$	0.5448	0.5448	0.5625	0.5625

Notes: dependent variable: ln(amount); treatment: same party. N does not include singletons. All regressions include month-times-year-, grant-recipient-, state- and ministry-fixed effects. Standard errors are clustered on state level; *p* values of wild bootstrapping with 9999 replications in parentheses

To get a better understanding of the exact mechanism, we examine four variations of the treatment variable. Table 6 has the results. First, Model (1) distinguishes between the two “sister parties” CDU and CSU. Although they form a parliamentary group in the German parliament, they are in a narrow sense two different parties, which we can examine separately. In addition, we focus on political preferences. To that end, Model (2) includes the variable “same political spectrum” which captures whether the federal minister and the state prime minister are from the same political spectrum. The left spectrum includes the SPD, the green party and the left party, while the right spectrum includes the CDU/CSU and the free democratic party (FDP). Furthermore, we consider the parties of the government coalition in the German Bundestag to be linked. The variable “coalition members” of Model (4) is equal to 1 if the state prime minister’s party is part of the government coalition in the Bundestag at the respective time. CDU and CSU were federal government parties for the entire observation period, but after the federal election in September 2013 the SPD replaced the FDP in December 2013. Finally, we analyze whether parties that are part of a state-level coalition but are not the prime minister’s party have an impact on project funding. The variable “part of state coalition” of Model (6) is equal to 1 if the federal minister is a member of a party that is part of the governing coalition of the state.

The coefficient of party affiliation when considering the two related parties CDU and CSU separately in Model (1) is positive, but smaller than when considering these parties together in the benchmark model and is only significant at the 10% level. This difference suggests that the two parties also support each other by funding projects.

Models (2) and (4) seem to suggest that members of the same political spectrum and of the federal government coalition are similarly aligned. However, Models (3) and (5) demonstrate that this is not true. These models compare membership of the political spectrum and the ruling coalition with membership of the same party. Model (3) uses “different political spectrum” as reference category and analyzes the effect of “same spectrum, different parties” and “same party” separately. In a similar way, Model (5) examines “different coalition members” and “same party.” Both the coefficients of “same spectrum, different parties” and “different coalition members” are not significant, whereas the coefficient of “same party” is significantly positive in Models (3) and (5).^15^ This demonstrates that the membership of the same party determines the spectrum and coalition effects. Since parties on the same political spectrum still pursue different political goals and compete with one another, and coalition members are only temporary allies, the political spectrum and coalition membership create weaker connections than party membership. While Model (6) indicates a positive effect of the party affiliation of the federal minister and the coalition members at the state level, Model (7) shows that this effect is mainly driven by the influence of the prime minister. Being represented in the coalition as a “junior” partner has a smaller, nonsignificant impact on project funding.

**Table 6:** Estimation results for variations of the treatment variable

	(1)	(2)	(3)	(4)	(5)	(6)	(7)
Same party (CDU $\ne$ CSU)	0.0284						
	(0.0795)						
Same political spectrum		0.0332					
		(0.0545)					
Same spectrum, different parties			− 0.0032				
			(0.8246)				
Same party			0.0400				
			(0.0540)				
Coalition members				0.0276			
				(0.0328)			
Different coalition members					0.0063		
					(0.6999)		
Same party					0.0447		
					(0.0137)		
Part of state coalition						0.0419	
						(0.0217)	
Junior in state coalition							0.0288
							(0.1181)
Same party							0.0436
							(0.0277)
*N*	82,249	82,249	82,249	82,249	82,249	82,249	82,249
$\hbox { R}^2$	0.5448	0.5448	0.5448	0.5447	0.5448	0.5448	0.5448

Notes: dependent variable: ln(amount). *N* does not include singletons. All regressions include month-times-year-, grant-recipient-, state- and ministry-fixed effects. Standard errors are clustered on state level; *p* values of wild bootstrapping with 9999 replications in parentheses

Table 7 shows results for both small and medium-sized projects as well as for all projects together (small, medium and large projects). Treatment coefficients are positive for small and medium-sized projects and for the universe of projects. But the coefficient is only significant for small and medium-sized projects, which indicates that the significance level decreases, the more we include larger projects. These results suggest that the link between party affiliation and funding gets weaker with increase in project magnitude. One explanation for this finding could be the more strictly regulated selection process for larger projects. It is likely that for larger projects stronger control mechanisms exist (Bundesministerium für Verkehr und digitale Infrastruktur, 2018) and there is also much more public participation to increase public acceptance (see, for example, Schmalz, 2019; Schönrock, 2019 and Umweltbundesamt, 2017), which presumably makes it more difficult to transfer resources according to the interests of the political parties. Since, in general, there are also more decision makers involved in decision processes on major projects, no politician alone can influence the allocation of funds. For example, in major projects, there often is involvement by overall project managers (Kompetenzzentrum (Groß-)Projektmanagement, 2020). For small projects, on the other hand, usually fewer control mechanisms exist, which in turn makes it more difficult to adequately monitor the allocation process.

**Table 7:** Estimation results for larger projects

	Small and medium projects	All projects
Treatment	0.0372	0.0312
	(0.0137)	(0.1513)
*N*	84,879	85,348
$\hbox { R}^2$	0.5174	0.5104

Notes: dependent variable: ln(amount); treatment: same party. *N* does not include singletons. All regressions include month-times-year-, grant-recipient-, state- and ministry-fixed effects. Standard errors are clustered on state level; *p* values of wild bootstrapping with 9999 replications in parentheses

### Estimation of changes into treatment

Table 8 shows the results of an estimate with lags and leads from changing into treatment for small projects, defined by Eq. (2). The reference category of the analysis is “currently no treatment, but change into treatment more than one month later or never in the future.” The effect of change into the treatment is greatest when it is 3 months ago (the more recent changes are not significant). It becomes significantly weaker if the change happened earlier. The non-statistically significant coefficients of future changes indicate that the ministerial bureaucracy does not independently anticipate the change. This can be interpreted as a sign of direct intervention by the head of the ministry or of the importance of management position changes.

**Table 8:** Estimation results for change into treatment

	ln(amount)
Treated now	0.0199
	(0.4563)
Treated one month ago	0.0922
	(0.1357)
Treated two months ago	0.2722
	(0.1054)
Treated three months ago	0.2379
	(0.0307)
Treated four months ago	0.2286
	(0.0349)
Treated five months ago	0.1579
	(0.0636)
Treated earlier	0.0299
	(0.0928)
Treated one month later	− 0.0087
	(0.8934)
*N*	82,249
$\hbox { R}^2$	0.5450

Notes: dependent variable: ln(amount); treatment: change from different parties to same party; reference category: change into treatment more than one month later or never. *N* does not include singletons. All regressions include month-times-year-, grant-recipient-, state- and ministry-fixed effects. Standard errors are clustered on state level; *p* values of wild bootstrapping with 9999 replications in parentheses

### Estimation results at the state-ministry level

We aggregate funding for every month and any combination of state and ministry, leading to 9600 ($=$ 10 years $\times$ 12 months $\times$ 16 states $\times$ 5 ministries) observations. For 2338 observations, there is no funding. We consider four outcome variables, a dummy variable “any project,” “funding per project,” “number of projects” and “ln(total funding).” For the last three variables, we include only observations with positive funding. We include month-times-year-, state- and ministry-fixed effects and cluster standard errors at the state level. Table 9 shows the results for estimating Eq. (3) for aggregated small projects. The treatment only has significant effects on funding per project; it does neither increase the likelihood of any funding nor, if there is funding at all, the number of projects and total funding. The estimated effect on funding per project is roughly 4/5 larger than the estimate we got at the project level.

These results suggest that project planners do not consider the political environment when submitting projects and that the political influence is primarily in the setting of the budget. While independent experts determine the eligibility of funding, administration and politicians can exert influence on the specific allocation of funds. That is not surprising, since the budget size and distribution is obviously not decided solely on the basis of scientific quality, but also on the basis of political and social objectives, as well as the budget constraint of the ministry.

**Table 9:** Estimation results for small projects aggregated at state-ministry level

	(1)	(2)	(3)	(4)
	Any project	Funding per project	Number of projects	ln(Total funding)
Treatment	$- 0.0043102$	17904.42	$- 0.4361712$	0.0755613
	(0.7985)	(0.0213)	(0.6362)	(0.1911)
*N*	9600	7262	7262	7262
$\hbox { R}^2$	0.3952	0.2565	0.7088	0.6779

Notes: treatment: same party (monthly lagged). All regressions include month-times-year-, state- and ministry-fixed effects. Standard errors are clustered on state level; *p* values of wild bootstrapping with 9999 replications in parentheses

## Conclusion

Our analysis adds additional relevant insights to the existing literature on distributive politics: In the literature, the focus often lies on regional assistance when addressing the question if politico-economic criteria play a role in grant allocation. Our paper shows that politico-economic criteria are also relevant in the geographical distribution funding for research, development and innovation projects, a topic which has not received much attention so far. A link between party affiliation and grant allocation is obviously existing in Germany for small projects, where control mechanisms are less strict than for major projects. These results allow to draw some interesting conclusions for grant allocation. In the public perception, major projects are often considered as involving inefficient allocation of resources. One reason for this view could lie in the fact that in many cases, major projects finally turn out to be much more expensive than originally planned, which is frequently perceived as a waste of resources. Although this might point to general problems concerning public procurement, for example, our analyses do not indicate that funding for major projects is considerably distorted by politico-economic criteria. While the link between party affiliation and grant allocation is weak for all projects, it is considerably stronger for small projects. So the issue of politico-economic aspects influencing grant allocation might be addressed by better supervising approvals of small projects, where control mechanisms might not be strong enough yet to prevent favoritism.

Although we only have access to a comparatively limited range of information in the entire process of project funding decision making, we can already measure a distorting effect of belonging to the same party on the funding of research, development and innovation projects. For future research, it would be very desirable to get information on the administrative and political mechanisms behind the approval decisions. In particular, it would be interesting to see if approvals of projects where political alignment exists are more or less likely if competitiveness among applicants increases. Moreover, additional information on the specific evaluation of the quality of applications according to certain defined criteria would also yield valuable insights. Do these criteria equally play a role in the final decision? Or are there some exceptions made in cases where, for instance, only one criterion is not fulfilled or one application is only slightly worse in one criterion to favor a certain application? Of course, these questions affect a highly confidential aspect of the project application procedure and thus, it is not too likely to get any additional information allowing to address these reflections.

The finding that there is a connection between party membership and granting of funding for research, development and innovation projects in Germany has important political implications. It implies that grant allocation is, at least in part, not only determined by objective quality criteria but somewhat distorted by criteria that do not contribute to efficient use of resources. In terms of assessing the efficiency of public spending on research, development and innovation projects, this means that less suitable applications could be given preference than those that would actually be best suited in terms of the official allocation criteria. Therefore, the efficiency of public spending could be increased if these distortions were reduced. This could possibly be achieved through greater transparency of allocation and funding or through delegation to expert commissions unrelated to the political parties. In any case, the issue of biases in the allocation of grants deserves further attention in the future. Efforts should therefore be made to provide access to more information on funding decision-making processes.

Finally, it would be important to find out to what extent the results can be transferred to other countries, also in order to better understand the role of specific institutions.

## Appendix

See Fig. 2 and Tables 10, 11, 12, 13, and 14.

**Table 10:** Structure of the data in the Funding Catalogue

Variable	Description
Ministry	Ministry that approved the funding
Grant recipient	Direct beneficiary of the funding
Executing entity	Entity where project is actually conducted
Topic	Description of the content of the project
Systematic classification	Field of research the project is related to
Beginning and end of project duration	Start date and end date of the project
Amount of assistance	Sum of funding in euro granted for the full project length
Funding profile	Broad category in which the project falls
Joint project	Information on whether there are several project partners having applied for a project together

**Table 11:** Top ten largest projects

Grant recipient	Amount of assistance
Deutsche Forschungsgemeinschaft (DFG) (German research association)	2,049,099,650
Facility for antiproton and ion research in Europe (FAIR)	766,289,553
Deutscher Akademischer Austauschdienst (German academic exchange service)	449,475,195
Deutsche Forschungsgemeinschaft (DFG) (German research association)	409,264,000
Alexander von Humboldt-Stiftung (Alexander von Humboldt-foundation)	289,917,529
Deutsche Forschungsgemeinschaft (DFG) (German research association)	288,750,000
Stiftung Begabtenförderung berufliche Bildung (SBB) (Vocational training foundation for the highly talented)	284,710,613
Fraunhofer-Gesellschaft (Fraunhofer society)	281,482,032
Stiftung Begabtenförderung berufliche Bildung (SBB) (Vocational training foundation for the highly talented)	236,903,691
Gauss centre for supercomputing (GCS)	226,333,333

**Table 12:** Classification of projects

	Small	Medium	Large	Mega / Program
Structure of project	Project leader (PL)-team	PL-team, overall-PL	Overall-PL, PL-team, project management office (PMO)	Program manager, overall-PL, PL-team, program office
Communication	Easy (PL)	Extensive (overall-PL)	Communication plan (overall-PL, PM, PMO)	Communication plan (separately for sub-projects)
Planning / Controlling	1 plan by PL	Overview/ detail by overall-PL + PL	Several sightings, PMO (determined function)	Map, determined function, separate sub-project
Project management (PM) processes	Mostly pragmatic; minimum of structure	Structured	Formal, support by determined function	Formal, complex, separate sub-projects
Total effort in persons/year (PY)	< 5	> 5 - < 50	> 50 - < 500	> 500
Total costs in million euro	< 2	> 2 - < 10	> 10 - < 100	> 100
Economic efficiency according to Federal Budget Code (BHO)	< 0,5 PY version 1(+4), < 5 PY version 1,2(+4)	All versions	All versions	All versions
Examples	Migration software	Introduction of document management system	Consolidation of distributed computer centers	IT-invest program / stimulus package II

Notes: dimensions of project classification provided by the Federal Office of Administration—Competence Centre (Major) Project Management. Our analysis relies on the classification according to project size, defined as total costs in million euro. Projects are defined as small if the total costs do not exceed 2 million euro. Medium-sized projects are those with costs between 2 and 10 million euro, major projects cost more than 10 million euro and mega projects are larger than 100 million euro. *Source:* (Kompetenzzentrum (Groß-)Projektmanagement, 2020)

**Table 13:** Variation in the share of projects across states and ministries

	(1)	(2)	(3)	(4)	(5)	(6)
State	BMBF	BMEL	BMU	BMVI	BMWi	Total
Baden-Württemberg	15.53	11.37	18.64	19.98	16.84	16.53
Bavaria	13.79	12.65	15.37	25.53	16.66	15.57
Berlin	8.58	5.31	2.35	3.08	8.27	6.79
Brandenburg	3.08	5.39	2.17	1.82	2.60	2.82
Bremen	1.81	1.09	0.78	0.77	2.09	1.54
Hamburg	2.48	1.41	1.02	1.98	3.38	2.27
Hesse	5.89	9.17	6.22	5.71	5.73	6.04
Lower Saxony	7.22	16.48	14.66	6.95	9.14	9.17
Mecklenburg-Vorpommern	2.23	3.88	1.84	2.70	1.64	2.18
North Rhine-Westphalia	18.40	12.35	17.83	14.42	17.73	17.57
Rhineland-Palatinate	3.10	4.55	7.65	3.43	2.18	3.85
Saarland	1.11	0.15	1.16	0.64	1.03	1.02
Saxony	7.99	6.92	1.36	6.51	7.34	6.54
Saxony-Anhalt	2.52	3.76	1.04	1.35	1.29	2.01
Schleswig-Holstein	2.52	3.09	6.54	2.89	2.02	3.21
Thuringia	3.74	2.45	1.38	2.26	2.07	2.88
Total	100.00	100.00	100.00	100.00	100.00	100.00

Notes: projects funded per state by the following ministries: (1) Federal ministry of education and research (BMBF), (2) Federal ministry of food and agriculture (BMEL), (3) Federal ministry for the environment, nature conservation and nuclear safety (BMU), (4) Federal ministry of transport and digital infrastructure (BMVI) and (5) Federal ministry for economic affairs and energy (BMWi). Column (6) shows the total share of projects per state. *Data Source:* Funding Catalogue (Bundesministerium für Bildung und Forschung - Referat Informationstechnik, 2020)

**Table 14:** Estimation results for small projects

	(1)	(2)	(3)	(4)	(5)
Treatment	0.0403	0.0566	0.0392	0.0421	0.0636
	(0.0426)	(0.1409)	(0.0148)	(0.0255)	(0.0064)
Month-times-year fe	Yes	Yes	Yes	Yes	Yes
State fe	Yes	Yes	No	No	No
Ministry fe	Yes	Yes	No	Yes	No
State-ministry fe	No	No	Yes	No	No
Recipient fe	Yes	No	Yes	Yes	Yes
State-election-period fe	No	No	No	Yes	No
State-ministry-election-period fe	No	No	No	No	Yes
*N*	82249	95782	82249	82249	82244
$\hbox { R}^2$	0.5448	0.3326	0.5466	0.5451	0.5501

Notes: dependent variable: ln(amount); treatment: same party. *N* does not include singletons. Standard errors are clustered on state level; *p* values of wild bootstrapping with 9999 replications in parentheses

## Footnotes

1 Studies investigating whether more governmental transfers are granted to home constituencies or home regions: For Belgium, see Jennes and Persyn (2015). See Stratmann and Baur (2002) and Maaser and Stratmann (2016) for Germany, Psycharis et al. (2019) for Greece, Carozzi and Repetto (2016) for Italy, Knight (2002, 2004) and Berry and Fowler (2016) for the US.

2 Studies exploring whether the location of core/swing voters impacts the distribution of grants: For France and Italy, see Dellmuth et al. (2017), Kauder et al. (2016) for Germany, Castells and Solé-Ollé (2005) for Spain and Ansolabehere and Snyder (2006) for the USA.

3 See Cadot et al. (2006) for evidence on France and Pinho and Veiga (2007) and Migueis (2013) for a maturing democracy, namely Portugal. Pinho and Veiga (2007) suggest that political alignment was especially important in years where the democracy was not yet fully established, but not in later periods.

4 See Colonnelli et al. (2020) for Brazil, Brassiolo et al. (2020) for Ecuador, Bach and Veit (2017) for Germany and Fiva et al. (2021) for Norway.

5 There are recent studies which focus on the regional perspective of the grants’ effectiveness. Cataldo and Monastiriotis (2020), for instance, show a positive effect of EU structural funds on economic growth in the UK overall and for less developed regions in particular. Kang et al. (2019) find that medical sector research grants in the USA seem to foster employment, especially in regions where intellectual capital is plentiful. Likewise, Tingvall and Videnord (2020) show for Sweden that publicly sponsored research and development grants have positive growth effects if channeled toward small and medium sized enterprises located in regions where skilled labor is copious.

6 Besides federal funding, there are a number of state-specific technology, research and innovation funding programs designed for German enterprises and institutions. Moreover, the European Commission offers support in these fields in the context of specific funding programs, like the European Cooperation in Science and Technology, Eureka, the Eurostars program or Horizon Europe.

7 Examples of federal institutions engaging in research and innovation activities and eligible for project grants are the Robert Koch Institute or the Federal Institute for Research on Building, Urban Affairs and Spatial Development. Fraunhofer-Gesellschaft, Max-Planck-Gesellschaft and the German Research Foundation are considered non-federal institutions. Besides (federal) institutions, private companies are an important innovation-elevator by contributing almost 70% of research and innovation expenditures. Especially activities by small and medium sized enterprises can be considered pathbreaking for regions’ innovation progress (Bundesministerium für Bildung und Forschung - Referat Grundsatzfragen von Innovation und Transfer, 2021).

8 See www.foerderinfo.bund.de/de/foerderfinder-1715.php for current published program proposals.

9 There are few projects included in the database with amounts of assistance of 0 euro, four projects with positive amount below 200 euro one of which is explicitly marked as an additional payment, and one project with a negative value of −82,210 euro described as “pseudo-project”.

10 A closer look at the distribution of projects revealed a high frequency of projects below 50,000 euro, the threshold below which the Federal Budget Act (BHO) permits exceptions for project financing. We also found descriptive evidence for bunching at certain round numbers, in particular, at 50,000 euro, 100,000 euro, 150,000 euro, 180,000 euro, 200,000 euro and 300,000 euro—some of which are upper limits for certain funding programs. We also analyzed funding around these thresholds, but did not find any systematic and consistent differences between treated and non-treated projects in this regard.

11 After the federal election in September 2017, it took more than 5 months for the coalition negotiations to be concluded, so the year in which a new federal government took office was 2018.

12 To reduce the number of symbols, we use the letter $\beta$ for our main coefficient(s) of interest and the letters $\eta$, $\gamma$, $\delta$, and $\lambda$ for fixed effects in different models.

13 Standard errors are typically larger than those based on cluster-robust variance estimators.

14 Since coalitions with several parties involved rule predominantly at federal and state level and we also do not use municipalities with many heterogeneous units as analysis units, we cannot identify core or swing voters appropriately and therefore do not include them in the analysis.

15 In Model (3), the significance level is marginally above 5%.
